# A novel undergraduate biomedical laboratory course concept in synergy with ongoing faculty research

**DOI:** 10.1002/bmb.21563

**Published:** 2021-07-22

**Authors:** Willemijn D. Schot, Maria A. Hegeman, Toine ten Broeke, Floris A. Valentijn, Irma Meijerman, Frans J. Prins, Wim J. A. G. Dictus, Niels Bovenschen

**Affiliations:** ^1^ Educational Consultancy & Professional Development, Faculty of Social and Behavioral Sciences Utrecht University Utrecht Netherlands; ^2^ Department of Pathology University Medical Center Utrecht Utrecht Netherlands; ^3^ Department of Pharmaceutical Sciences Utrecht University Utrecht Netherlands; ^4^ Center for Academic Teaching Utrecht University Utrecht Netherlands; ^5^ Center for Education University Medical Center Utrecht Utrecht Netherlands; ^6^ Center of Translational Immunology University Medical Center Utrecht Utrecht Netherlands

**Keywords:** authentic research, inquiry‐based learning, research‐based learning, students as partners, undergraduate

## Abstract

Optimal integration of education and ongoing faculty research in many undergraduate science programs is limited to the capstone project. Here, we aimed to develop a novel course‐based undergraduate research experience (CURE) in synergy with ongoing faculty research. This 10‐week course called Biomedical Research Lab is embedded in the curriculum of the undergraduate program Biomedical Sciences and grounded in the theoretical framework of research‐based learning. Four groups of four students work together in a dedicated laboratory on an actual ongoing research problem of faculty. All groups work on the same research problem, albeit from different (methodological) perspectives, thereby stimulating interdependence between all participants. Students propose new research, execute the experiments, and collectively report in a single research article. According to students, the course enhanced scientific, laboratory, and academic skills. Students appreciated ownership and responsibilities of the research, laboratory teachers as role models, and they were inspired and motivated by doing authentic actual research. The course resulted in a better understanding of what doing research entails. Faculty valued the didactical experience, research output and scouting opportunities. Since topics can change per course edition, we have showcased a widely applicable pedagogy creating synergy between ongoing research and undergraduate education.

## 1 INTRODUCTION

To prepare students for the demands from the labor market, universities pay much attention to train students the required academic skills. Among others, these include communication, critical thinking, creative thinking, (interdisciplinary) problem solving, collaboration, project management, and self‐organization skills.^1^ Academic skills and deep‐learning of many students prosper in a didactic framework of constructivism^2^ and research‐based education.^1^, ^3^, ^4^, ^5^, ^6^ This latter pedagogy is generally considered as a research‐minded, student‐centered approach, based on learning by addressing relevant questions and complex authentic research tasks.^1^, ^3^, ^4^, ^5^ Students elaborate on real‐world actual research and actively learn the academic skills to facilitate a better transition to master programs and the labor market.^7^ Working with role models further enhances student motivation and inspiration.^4^, ^8^

Research‐based education is grounded in a widely accepted framework developed by Griffiths^9^ and further shaped by Healey (2009). This framework expresses two axes, one with the dimensions “teacher‐focused” and “student‐focused,” and the other one with “research content” and “research processes/problems.” The resulting quadrants form four scenarios that describe the relationship between teaching and research: (a) Research‐led: where students learn about research findings and information transmission is the main teaching mode, (b) Research‐oriented: where students learn about research processes, including state‐of‐the‐art technology, (c) Research‐tutored: where students learn to discuss and write research papers, and (d) Research‐based: where students learn as researchers with inquiry‐based activities and the research‐cycle, including doing hands‐on research.^4^ While all quadrants are important in curriculum design, the latter two student‐focused quadrants are often limited and underrepresented in educational undergraduate science programs.^7^ This is a missed opportunity, since in these quadrants students most actively participate in research.

Next to the positive effects of research‐based education on student learning, it has been well established that scientific research can also benefit from teaching.^8^, ^10^ Teaching tasks force researchers to hold a broad overview and perspective on their discipline, lead to better reflection on their research, and raise talent scouting opportunities.^11^ Students can generate new research questions and hypotheses, give feedback and new ideas, and enhance (societal) relevance by continuing to ask (global) questions. Students generate new data and insights that can be beneficial for researchers.^11^ However, apart from the classical bachelor thesis (capstone project), creating synergy between research and teaching to enhance the research‐teaching nexus in undergraduate programs often does not occur naturally and this relationship even has the tendency to diverge due to political and institutional policies and cultures.^3^, ^10^

In the present study, we aimed to develop a novel course‐based undergraduate research experience (CURE) that creates strong synergy with ongoing faculty research, in which scientists and students are seen as partners. All students work in subgroups on the same research problem of a faculty, albeit from different methodological perspectives. Within the boundaries of this research problem, students propose hypotheses and the research, execute the experiments and collectively interpret and report their data. We evaluated this course at the levels of technical (laboratory) skills, academic skills, views and attitudes toward science, the research‐teaching synergy, and the effects of following this course on the bachelor thesis (undergraduate capstone project). This widely applicable novel educational CURE concept not only enhances student academic and scientific skills, but also fosters ongoing faculty research.

## 2 COURSE DESIGN

### 2.1 Educational environment

**FIGURE 1:**
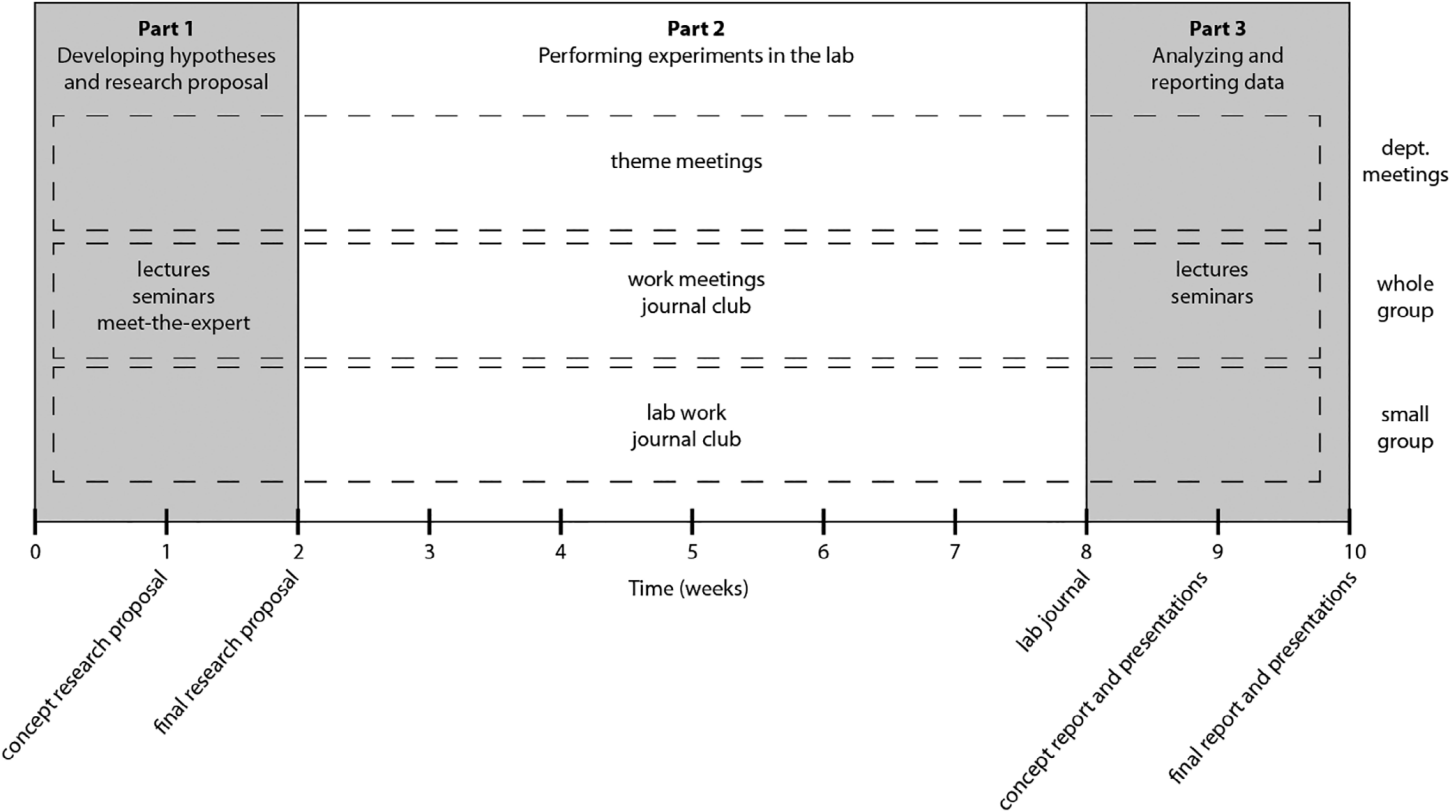
Set‐up of the course Biomedical Research Lab

In the undergraduate program Biomedical Sciences (approximately 175 students per year) at the faculty of Medicine, Utrecht University (Utrecht, The Netherlands), each academic year is divided into four equal periods of 10 weeks that each harbor 1–2 courses. Our novel Biomedical Research Lab (BRL) course is a full‐time 15 ECTS (European Credits Transfer System) elective course positioned in the second and third period of the third year, with a maximum of 16 students per course (Figure 1). In the event that more than 16 students want to enroll in a BRL course, students are selected via allotment. Alternatively, students can choose among several other elective courses in the same period. All students had basic text‐book knowledge of molecular biology, cell biology, physiology, and research methods, as well as basic laboratory skills (standard cook‐book practicals) during year 1 and 2 courses.

In the BRL course, all students get the opportunity to participate in one authentic ongoing research project from faculty (principal investigator) of the Faculty of Medicine at Utrecht University. The current study describes the first four consecutive editions of the course that were offered in 2017–2020. Each course edition highlighted a different scientific theme, that is, virus immunology (2017–2018, period 2), tumor immunology (2018–2019, period 2 and 2019–2020, period 3), and oncology (2019–2020, period 2). Main aims of the course are clustered in knowledge, skills, and attitudes, including training of scientific and academic skills, completing the research cycle with hands‐on research and dealing with the uncertainties of experimental results. The specific learning goals are listed in Table 1.

**TABLE 1:** Learning goals of the course Biomedical Research Lab

Domain	After this course students are able to:
Knowledge and insights	Explain the most important concepts and theories of the subject of study.
	Integrate and discuss these concepts and theories: predict experimental results based on theory, develop theory based on experimental results to contribute to new scientific insights.
Skills	Find and critically evaluate scientific literature.
	Formulate (sub‐)hypotheses based on scientific literature and ongoing (unpublished) research in the faculty.
	Determine methods to approach the research question (from various angles).
	Use lab techniques to obtain experimental data to answer the research question.
	Draw conclusions based on the data and scientific literature.
	Analyze, combine, and integrate the data to apply it to a scientific discussion.
	Present the study in a scientific article.
	Present the study in an oral presentation.
	Formulate the (societal) relevance of the study.
Attitude	Take responsibility for their research.
	Cooperate to obtain the best possible group outcome.
	Be critical toward themselves and other students.
	Keep to the rules of the laboratory.
	Process the results with scientific integrity.

The laboratory costs (bench fees) and part of the supervisor salaries were financially compensated by the standard educational compensation fee of the program Biomedical Sciences of Utrecht University.

### 2.2 Content and design

The course consisted of three separate parts; (a) Defining hypotheses and writing research proposals (week 1–2), (b) performing experiments in the lab (week 3–8), and (c) analyzing and reporting data (week 9–10).

#### 2.2.1 Part 1: Defining hypotheses and writing research proposals

At the start of the course, a principal investigator introduces the subject and poses the research problem that is cutting‐edge and topical in the faculty's lab. This problem is embedded in literature, which was provided to the students through scientific papers and lectures, including unpublished data. During the first 2 weeks of the course, students framed hypotheses and wrote a proposal for the research they would perform in the next part of the course. For this, the 16 students were divided into 4 groups of 4 students each, resulting in 4 hypotheses and covered by 4 research proposals. Importantly, all groups addressed the same overall research problem, albeit from different (methodological) perspectives. Staff members were readily available for moderation and questions during the proposal conceptions and through planned meet‐the‐expert sessions (1 h every day). Moreover, tutorials were offered on skills, i.e., how to keep a lab journal, basics for writing a research proposal and how to write an introduction of a scientific article. Each group wrote their concept proposal including an introduction section in week 1 (max three pages), gave and received feedback from peers and faculty on the proposals, and delivered a pitch of the final version of their proposal at the end of week 2. The four introduction sections (1 per group) were used later in phase 3, writing a research article. Part 1 of the course ended with a tour in the research lab where they were going to perform their proposed experiments in the next phase. Nearly all contact time in part 1 was provided by a principal investigator supported by his/her research staff, including PhD students, postdocs, technicians and (under)graduate students.

#### 2.2.2 Part 2: Performing experiments in the lab

In the second part of the course (weeks 3–8), students performed their proposed experiments in a well‐equipped research laboratory at the University Medical Center Utrecht (UMCU) that was fully assigned to the students. When necessary, more specific techniques were done in the lab of the principal investigator or in core facilities at the UMCU. Each group of four students was guided by a supervisor affiliated with the laboratory of the principal investigator. In the 2017–2020 editions of the course, the lab supervisors were composed of mixtures of a postdoc, a PhD student, a senior lab technician, a graduate student, or undergraduate student. Experiments performed by the students were directly relevant to the research of the postdoc, PhD student, or PI. In addition to lab work, students participated once a week in (a) a journal club, in which students selected a scientific article that they presented and discussed with their peers and supervisors, (b) a general theme meeting, in which PhD students and postdocs of the UMCU presented their work (only for course editions 1, 2, and 4), and (c) a work meeting in which students presented the (raw) data obtained from their experiments to peers, supervisors, and staff member. At the end of part 2, students handed in their lab journals.

#### 2.2.3 Part 3: Analyzing and reporting data

The last 2 weeks of the course, students analyzed and reported their data. There were two tutorials covering writing a scientific article and preparing an oral presentation. Whereas all four groups of students prepared their own final presentation, all 16 students were reshuffled in new groups, each addressing one particular section of a full scientific manuscript. As such, all students, supervisors, and the principal investigator worked together on the same final end product of the course, a scientific article. For this, one student was appointed as coordinator of the writing process. The introduction section of the manuscript was extracted from the four proposals as written in phase 1. After 1 week, the students gave and received peer feedback on the concept version of their scientific article. Also, they practiced their oral presentations together with the faculty member. On the final day of the course, students gave their oral presentations to their peers, lab supervisors, faculty member, the course coordinator, and other interested faculty. In addition, they handed in the final version of their scientific article.

### 2.3 Assessment

To pass the course, students needed to obtain a passing grade (5.5 on a 10‐point scale) on all of the following assessment criteria: Performance during lab work (20%), Final written scientific report (20%), Final oral presentation (20%), Lab journal (20%), and General effort (20%).

### 2.4 Supervisor training

All faculty and supervisors received an educational training from experienced educational specialist (author MAH). Prior to the BRL course, a 4‐h workshop was provided in which supervisors were taught how to guide undergraduate students in a lab, with a particular focus on giving feedback and how to motivate students. During the BRL course there were three moments of peer consultation. In addition, when master students were involved in the guidance, they were offered the opportunity to reflect on their educational development in a thesis. For this, they were awarded with nine ECTS (educational internship).

## 3 COURSE EVALUATION

The study is approved by the Ethics Committee of the Faculty of Social and Behavioral Sciences of Utrecht University. The approval is based on the documents send by the researchers as requested in the application form of the Ethics committee and filed under number 20‐506.

### 3.1 Written questionnaire

All participating students (*n* = 53) from the four editions of the BRL course completed a written questionnaire at the end of the course. The results are displayed in Table 2. Most items were scored on a 5‐point Likert scale (−−, −, ±, +, ++), with the exception of the items “the level of this course was” and “the required time investment was.” These items were rated from “much too low (score 1)” to “much too high (score 5).” Finally, students were asked to rate the course as a whole on a 10‐point scale (a 10 being the highest rate) and there were comments fields where students could make open remarks related to learning activities, supervision, workload, and any remaining issues.

**TABLE 2:** Post‐course student evaluation

Course edition	2017–2018		2018–2019		2019–2020		2019–2020	
	(Period 2)		(Period 2)		(Period 2)		(Period 3)	
	*n* = 16		*n* = 16		*n* = 16		*n* = 5	
Survey items	M	*SD*	M	*SD*	M	*SD*	M	*SD*
*Content and organization*								
This course fitted well with my prior knowledge	**4.2**	0.5	**4.3**	0.8	**4.3**	0.4	**4.4**	0.5
I was informed well about this course	**4.3**	0.6	**4.2**	0.5	**4.3**	0.6	**4.6**	0.5
The course was well designed	**4.4**	0.5	**4.5**	0.5	**4.6**	0.5	**4.6**	0.5
I obtained a lot of knowledge during this course	**4.7**	0.6	**4.9**	0.4	**4.9**	0.3	**5.0**	0.0
I was able to explore doing scientific research during this course	**4.8**	0.6	**5.0**	0.0	**4.9**	0.3	**5.0**	0.0
My enthusiasm for scientific research increased during this course	**4.4**	0.7	**4.1**	0.6	**4.4**	0.6	**5.0**	0.0
The course was scheduled well	**3.9**	0.6	**3.9**	0.5	**4.3**	0.7	**4.4**	0.5
The rooms for this course were adequate	**4.1**	0.9	**4.2**	0.4	**4.1**	0.7	**4.6**	0.5
The required time investment was^a^	**3.5**	0.5	**3.5**	0.5	**3.1**	0.3	**3.0**	0.0
The level of this course was^a^	**3.2**	0.4	**3.2**	0.4	**3.2**	0.4	**3.4**	0.5
*Learning activities*								
I learned from conceiving the hypotheses (part 1)	**4.3**	0.5	**4.1**	0.4	**4.2**	0.4	**4.0**	0.0
I learned from writing the research proposal (part 1)	**4.3**	0.5	**4.1**	0.3	**4.3**	0.5	**4.6**	0.5
I learned from performing the research in the lab (part 2)	**4.9**	0.3	**4.8**	0.4	**4.9**	0.3	**5.0**	0.0
Working on actual, relevant, ongoing research was motivating and inspiring.	**4.7**	0.6	**4.7**	0.5	**4.8**	0.4	**4.6**	0.5
I learned from keeping a lab journal (part 2)	**4.0**	0.8	**3.5**	0.7	**4.0**	0.7	**4.2**	0.4
The group size (four students per sub‐hypothesis) was adequate	**3.9**	1.0	**4.0**	0.5	**4.5**	0.6	**N/A**	N/A
I learned from the work meetings (part 2)	**4.1**	0.6	**3.9**	0.6	**4.1**	0.8	**4.8**	0.4
I learned from the journal club (part 2)	**2.9**	0.7	**3.3**	0.7	**3.8**	0.8	**4.4**	0.5
I learned from the Immunology theme meeting (part 2)	**3.6**	0.8	**3.4**	0.7	**N/A**	N/A	—	—
I learned from writing the scientific report (part 3)	**4.5**	0.5	**4.3**	0.6	**4.4**	0.6	**4.6**	0.5
I learned from giving the oral presentation (part 3)	**4.1**	0.6	**4.1**	0.6	**4.2**	0.7	**4.0**	0.0
During this course I improved my Academic skills	**4.5**	0.5	**4.5**	0.5	**4.6**	0.5	**4.4**	0.5
*Supervision*								
The teachers were enthusiastic and involved	**4.7**	0.5	**4.6**	0.5	**4.9**	0.4	**5.0**	0.0
The teachers were knowledgeable	**4.5**	0.7	**4.2**	0.4	**4.7**	0.5	**5.0**	0.0
The daily supervision in the lab was adequate	**4.5**	0.5	**4.3**	0.6	**4.9**	0.4	**4.8**	0.4
My fellow students put in their best effort	**4.3**	0.5	**4.4**	0.5	**4.7**	0.5	**4.8**	0.4
*Overall*								
There was a good atmosphere during the course	**4.6**	0.5	**4.6**	0.5	**4.8**	0.4	**4.6**	0.5
I give this course the following grade (10 point scale)	**8.8**	0.4	**8.6**	0.6	**8.9**	0.4	**9.2**	0.4

*Note*: Likert scale rating from 1 (“I highly disagree”) to 5 (“I highly agree”).

Abbreviations: M, mean; N/A, not applicable; *SD*, standard deviation; −, response rate insufficient.

^a^ These items were poled from “much too low (score 1)” to “much too high (score 5).”

Bold values represent means of course evaluations.

Students appreciated the BRL course as is evident from the high scores on the statements (Table 2), the high grade for the course in general (8.6–9.2; 1–10 point scale), and the answers to the open questions in the written evaluation. Students appreciated going through the research cycle, ranging from framing a research proposal to executing the experiments and interpreting and reporting their data. One student mentioned: “*working on ongoing new research and coming up with the hypotheses and experimental design ourselves was fun and I learned a lot from it*. *I also learned a lot because we were allowed to do almost all the lab work ourselves*” (2017), and “*I appreciate the free choice in your own research and developing your academic skills*” (2019). Students found the BRL course inspiring and motivating, thereby enhancing academic skills: “*I found executing current and our own proposed research so much more motivating compared to pre‐arranged ‘cookbook’ practicals*” (2018), “*An opportunity to really deal with research and the feeling that you are taken seriously as an undergraduate stude*nt” (2019), and “*A course where you perform research within a current ongoing investigation*, *while working fanatically for 6 weeks*, *was very motivating for me*. *I would recommend this course to everyone that wants to experience working in a lab*” (2020). Students also indicated that the BRL course gave them a more realistic view of doing research. Students wrote “*this course is really close to research*. *I really know much better what research entails thanks to this course*” (2017), and “*It really gives a sneak preview and a good preparation for future graduate internships*. *Also appreciated the supervision*, *very easy to reach and knowledgeable*” (2020). Finally, some students made comments about the time investment of the course, stating that it was high but that they knew this in advance and that it was necessary and worth it.

The most important points of improvement that the students initially mentioned in 2017–2018 were related to (a) the structure of the journal club with statements such as “*the journal club was a good initiative and also important but as it was organized now*, *there was too little time to read and prepare all the articles*,” (b) combining all the research done in the course into one paper with statements such as “*I think it is better to write four separate papers because writing one paper with sixteen people is too chaotic*,” and (c) adjusting the group size or the activities within each group with statements such as “*a group of four people was too much in our case*. *We couldn't really divide the practical work*. *Therefore*, *at times two to three people had nothing to do*.” These comments prompted us to substantially improve these aspects (a–c) of the course toward 2020. (a) Students prepared and discussed a research paper on a weekly basis during the journal clubs in the 2017–2018 edition. This took too much preparation time while also performing lab experiments. Therefore, in 2019 we implemented one collective journal club in part 3 of the course according to the “jig‐saw” method.^12^ In this method, students had 6 weeks (part 2) to search, read, and make a presentation of one article per group, after which groups were reshuffled to discuss the four papers with an extra focus on how to use this article in the discussion section of their final paper. Students appreciated this form of the journal club better, as was supported by the mean score for the statement “I learned from the journal club (part 2)” that gradually increased from 2.8 in the first edition to 4.4 in the latest edition. (b) Students of the 2017–2018 course edition also noted that it was quite a challenge to write one professional scientific article with 16 people in part 3 of the course. This concern was addressed in the next courses by appointing one student as “paper coordinator,” which provided much more structure for the writing process. (c) Students experienced that it can be difficult to divide and plan the work efficiently in a group of four people during part 2 of the course. Therefore, in subsequent BRL courses we stressed in advance that waiting times are inherent in doing research and that time‐management is an important academic skill to organize the workload better within their own subgroup. These approaches, in which students were given more autonomy to direct and influence their course activities, led to much more positive feedback on these issues during the years (Table 2).

### 3.2 Focus groups

Next to written evaluations, we conducted two focus groups with the students that followed the first edition of the BRL course in the academic year 2017–2018: one immediately after the BRL course and one after completion of their bachelor thesis (capstone project) in the fourth period of the third year (academic year 2017–2018). The latter was aimed at gaining further insight into how the course contributed to the students' technical and academic skills, their views and attitudes toward science, and their performance in the capstone project. In addition, we conducted a focus group with the supervisors of BRL course edition 2017–2018 to investigate how they experienced the supervision. The focus groups were led by authors WDS, MAH and IM, who did not teach or supervise the students in the BRL course. All students and supervisors gave informed consent prior to participation to the focus groups.

#### 3.2.1 Technical and academic skills

When asked about the research skills the students gained through the BRL course, they listed a wide range of skills covering the entire empirical cycle, including critical thinking, problem solving, collaboration, writing and pitching their idea, planning the experiment (especially regarding the logistics of ordering materials and incorporating factor time between subsequent steps), gathering and keeping track of the data in lab journals and reporting results ethically and understandable for the readers. Group cohesion was mentioned as a positive side effect of exchanging ideas and problem solving. Because all student groups worked on the same question with different lab techniques, students appreciated that one group could solve the problem of another group from a different perspective. Students also indicated that the BRL course made them more critical on research papers they read due to their experience with the journal clubs. Moreover, they focused more on the details and the required statistics in literature: “*before*, *I would have never noticed that a control was missing from an experiment*.” Writing the scientific article also made the students more critical about their own data: “*what data is still missing*?” and it made them aware that researchers need to “*sell the story at conferences and to get grant money*.”

#### 3.2.2 Views and attitudes toward science

Students indicated that the main reasons for signing up for the BRL course were gaining research experience and finding out whether research was something they would like to pursue in their Master and further careers. This means that the students that participated in the course actively sought out the opportunity to investigate their own views and attitudes toward (doing) science. In the focus groups, students mentioned that the BRL gave them a more realistic view of what doing science actually is. They specifically mentioned that doing research takes much more time then they anticipated as illustrated by these statements made by the students: “*when I read a textbook*, *I always thought I can do that too and very quickly*. *Now I know it's much harder than that*,” “*I know now that a single paragraph in a paper can be weeks of work*,” “*many things can go wrong*, *an article in a journal only shows the pretty picture*,” “*I understand now why obtaining a PhD takes four years*.” In addition, students indicated that joining research meetings and journal clubs gave them a good understanding of the collaborative nature of a research group. They did feel, however, that they collaborated more than in an average research group, because they worked in groups of four. In one of the focus groups, the students agreed on the idea that “*in real life*, *our group of four is probably like one researcher but with extra hands so it was easier for us to divide the work*.”

#### 3.2.3 Performance in the capstone project

The Bachelor Thesis (capstone project) of the Biomedical Sciences program at Utrecht University has a stronger emphasis on literature review than on lab work (8 weeks literature review and writing and 2 weeks lab work). When we asked students about the influence of attending the BRL course on their performance in the capstone project, students initially mentioned the technical lab skills they acquired during the BRL course. Students also mentioned other skills, such as searching for and synthesizing literature, placing your research within the literature and thinking about the implications and follow‐up questions of your research. Students indicated that they were more skilled in reading scientific literature and keeping lab journals. In addition, students mentioned that their experiences in the BRL course were very valuable when writing a scientific article during their capstone project. They specifically mentioned “*making choices and limiting what you present to your reader*” and “*being brief and concise when presenting your data*” as learning gains from the BRL that they could transfer to their capstone project. Finally, the students mentioned that participating in the BRL course had advantages in other courses because they had a better understanding of lab techniques, including those that lecturers mentioned during talks in the theme meetings about their own research.

#### 3.2.4 Research‐teaching synergy

In the BRL course, students contributed to relevant real‐world research and performed complex authentic research tasks under supervision of faculty as role models. We hypothesized that this would lead to a synergistic symbiosis between researchers and students. Indeed, we found that both students and faculty mutually benefited from the course.

In the focus groups with the students, they indicated that working on real‐life research was motivating because it “really mattered.” They compared the BRL course to “cookbook practicals” which are quite common in the biomedicine undergraduate program and found the BRL course more motivating and inspiring because the outcome of the research was unknown in advance. In addition, they stated that they learned more because when things did not go as expected, they had to think of solutions to the problem rather than see what the outcome of their neighbor's experiment was and continue from there. Students stated that the BRL course also had added value compared to coming up with “fictional experiments” because they had to consider constraints such as time, money, and the availability of resources.

In the focus group with the supervisors, they too mentioned that they felt that the real‐life experience and the enthusiasm of the students as well as the supervisors made the BRL course an inspiring and motivating learning environment. This made supervising within the BRL a fun experience well worth the time investment. Importantly, in addition to the learning gains they saw for the students (in line with those described by the students themselves), the supervisors also indicated that they themselves benefitted from supervising in the BRL course. For instance, the supervisors got an opportunity to supervise students and strengthen their didactical skills as they needed to explain the theory behind the experiment the students performed. The fact that they could do so adequately boosted their self‐confidence. Also, the data the students gathered lead to research output for the faculty members and working with the students gave the opportunity to scout excellent students for future intern positions. PhD Student (supervisor): “*The data the students generated are incorporated in a research article of my thesis and will be further worked out as a research paper for publication in a scientific journal*. *This research otherwise could not have been done*.” Supervisor/PI: “*Students that followed the BRL course know better what research in my lab entails and are therefore able to make a better informed decision to do a future Master internship in my lab*.”

Both the students and the supervisors emphasized the importance of the supervision in the success of the BRL course. The students specifically mentioned patience, trust, enthusiasm, optimism and creating a safe learning environment in which students are allowed to make mistakes, as stimulating characteristics of the supervision. They also see their supervisor as a role model of whom you can copy behaviors and skills. The supervisors mention that an important skill for example is to clarify the theory behind the experiments and explaining procedures and techniques to the students.

## 4 DISCUSSION

In this study, we developed a novel undergraduate educational concept that integrates ongoing faculty research with teaching. This concept harbors the classical hallmarks of a course based undergraduate research experience (CURE), including (a) scientific practices, such as asking questions, proposing hypotheses, designing studies, collecting and analyzing data, and communicating results, (b) development of new knowledge, (c) relevant or meaningful work, (d) collaboration, and (e) iteration.^13^, ^14^, ^15^, ^16^ Additive and, to our knowledge, new in our concept is that all 16 students work on the same research problem that comes from ongoing research of a faculty, and that students address this problem in subgroups from different scientific perspectives. This strengthens the research‐teaching nexus by creating mutual incentives and benefits for students and researchers that are multipronged.

All elements of Healey's framework of research‐based education^4^ are represented in the BRL course. Students learn about research (research‐led) and research processes (research‐oriented) in lectures, theme meetings, and journal clubs. Students also learn by doing in that the BRL course puts a strong emphasis on “research‐tutored” and “research‐based” elements, that is, writing/peer‐review and going through the research cycle, respectively.^4^ These latter two inquiry‐based and student‐centered elements are often poorly developed and underrepresented in undergraduate science programs.^7^ To this end, we have created a learning environment that is facilitated by a specific well‐equipped and dedicated laboratory that is assigned to the students. This recently founded *Bachelor Research Hub* is positioned in the middle of the research laboratory center at the University Medical Center Utrecht, with short lines to ongoing research and facilities.^17^, ^18^ Here, undergraduate students can meet other students, researchers, clinician‐scientists and medical doctors, and have the opportunity to do research both in course‐based research within the curriculum (i.e. BRL course) or extracurricular.^18^

We investigated how the BRL course affects students' academic skills and their perception toward science. Student data revealed that students gain a better perception of the research cycle. This ranged from dealing with details in experimental logistics up to placing their work in a broad and societal context. According to the students, this has significantly improved their academic skills. Students valued the autonomy and trust that were given thereby enhancing their motivation, which is compatible with the self‐determination theory of motivation.^19^ This theory states that autonomy, together with a feeling of competence and relatedness, fosters deep learning and academic skills.^19^ Students recognized the authenticity of their own real research and felt part of a larger research team.^20^ Interestingly, students found the group strategy and organization within the BRL course to be stimulating and constructive as many problems could be solved either within the individual student group or the whole groups, while working toward an answer to the common research question. This fits with the concept of interdependence and collaborative learning.^21^ Our findings are compatible with other more traditional CUREs that report gains in research skills such as critical thinking, problem‐solving skills, data analysis, and oral and written communication, as well as personal development skills such as self‐confidence and self‐efficacy.^20^, ^22^, ^23^, ^24^, ^25^, ^26^ CURE student' perceptions of collaboration and making relevant novel discoveries are positively related to their cognitive and emotional ownership, and impact on clarifying students' career intentions.^27^, ^28^ Moreover, CUREs stimulate learning a topic in depth, learning to work independently, building tolerance for obstacles faced in the research process, and transforming the student‐teacher relationship.^23^, ^24^, ^25^, ^26^ In the future, further interdisciplinary intra‐ and inter‐group cohesion and interdisciplinary dynamics could be an important addition to the BRL course. Collaboration with other disciplines can be a significant addition to the set of academic skills that are important for students' preparedness for their careers in academia and industry.^18^, ^29^, ^30^

Our study indicates that the BRL course concept enhances the synergy between research and teaching. On the one hand, the course format is successful in shaping research attitudes and transferring the major academic research skills to undergraduate students that are valuable for a better transition to master programs and labor market. This already becomes apparent in the students' progress during the capstone projects later in the same academic year, in which students realized advantages of skills developed during the BRL course. On the other hand, faculty researchers benefit by acquiring new data output, lots of relevant and critical questions and insights from students, financial support for laboratory costs and guidance, and scouting opportunities of excellent students for future research (internship) positions. Moreover, faculty, PhD students and even Master students benefit from training by educational specialists and the Master students get credited (ECTS) for participating as supervisor. Such a teaching experience for PhD and master students tends to improve their preparedness for a research career.^31^ Thus, we have showcased a mutually rewarding interplay between ongoing research and undergraduate education that is not only applicable to life sciences, but also constitutes a basic pedagogy applicable to other disciplines.

## CONFLICT OF INTEREST

N.B. was involved in the development of the course. N.B. and T.B. were involved in teaching in the course. N.B. was involved in the data analysis and writing of the research paper. None of the authors have any commercial interest in the work.
